# Supplementary material for: Seroepidemiologic survey of emerging vector-borne infections in South Korean forest/field workers
Source: PLoS Negl Trop Dis. 2021 Aug 18;15(8):e0009687. doi: 10.1371/journal.pntd.0009687 (PMC8405005; doi:10.1371/journal.pntd.0009687)
Supplement: Attachment — Submitted filename: Responses to Reviewers_TBE.docx [file pntd.0009687.s001.docx]

Responses to reviewers

Reviewer #1: The manuscript entitled “Seroepidemiologic survey of emerging vector-borne infections in South Korean forest/field workers” describes the sero-epidemiological feature of SFTSV and TBE. However, it didn't deviate much from showing predictable data. So, I recommend that authors would rather contribute to another suitable journal.

→ Thank you for your time and effort for the review of our manuscript.

TBEV is an important viral agent of the central nervous system infections in European countries, northern China, Mongolia, and Russia. The World Health Organization recommends vaccination against TBEV to all age groups in highly endemic areas of TBE, in which the average pre-vaccination incidence of TBE is ≥ 5 cases/100,000 persons per year.

In South Korea, no human cases of TBE have been reported so far, however, TBEV was detected from ticks and wild rodents. Due to the lack of reported human infection cases of TBE, awareness of physicians about TBE is very low in South Korea, leading to inactive performance of diagnostic tests.

This study would have a value providing some evidence of TBE, emerging vector-borne infectious disease, in South Korea. In addition, TBE vaccines are not introduced in South Korea and SFTS vaccines are not available to date. This study also provides epidemiologic data of neglected vector-borne diseases and would contribute to establishment of vaccine policy.

We are respectfully looking forward to your positive consideration about our manuscript.

Reviewer #2: The manuscript submitted by Jiyun et al describes seroepidemiologic survey of emerging vector-borne infections in South Korean forest/field workers, and provides the evidence of SFTSV and some flaviviruses potential exposure. The manuscript should be published after revision. Some of the helpful suggestions and comments are below.

→ Thank you for your time and helpful suggestions about our manuscript. We revised our manuscript as your comments. Point-by-point responses to your review are described below.

We really appreciate your positive consideration about our manuscript.

Please unify the positive rate on line 50…“0.1% (1/583)” and line 168…“0.2% (1/583)”, both for “0.2% (1/583)? or 0.17% in all the statement about SFTSV seropositivity in this paper.

→ SFTSV seropositivity was 0.2% (1/583) and we would like to present the proportion to one decimal place. We corrected the positive rate on line 50.

Line 50: If the seroprevalence including the positivity of neutralization, ELISA, and IFA, please re-describe it as TBEV and SFTSV were 1.03 % (6/583) and 0.17% (1/583).

→ Thank you for your nice comment. In author summary section, we would like to present seroprevalence of TBEV and SFTSV measured by neutralization assay. We described the seropositive rates of neutralizing antibodies for TBEV and SFTSV as 0.3% (2/583) and 0.2% (1/583), respectively (line 49-50).

Line 92 and 100: Please provide the strain or lineage information of TBEV and SFTSV.

→ For the TBEV neutralization assay, Neudörfl strain, a Western (European) subtype of TBEV, was used. For the indirect immunofluorescence assay (IFA) and plaque reduction neutralization test (PRNT) against SFTSV, clinical isolate from a PCR-confirmed SFTS patient was used. We provided the strain information of TBEV and SFTSV in line 92 and 101-102.

Line 121-123: Author described that one forest worker in Hongcheon showed very low neutralization titer (1:10) against TBEV, also, anti-TBEV IgG was not detected using ELISA in this person”, is this serum also negative for TBEV IgM? Can't you be able do IFA with TBEV infected antigen slides?

→ We conducted a cross-sectional investigation of seroprevalence of TBEV among forest/field workers and the study subjects were asymptomatic at the moment of investigation. Thus, we did not examine anti-TBEV IgM in this study. IFA for TBEV was not available for us, however, we measured neutralizing antibodies against TBEV. We added this point as a study limitation in line 205-206.

Line 184: Why the neutralization test for SFTSV was not performed? Because you don't have the SFTSV strain? If no, how you can able to prepare antigen slides infected with SFTSV in line 100?

→ Thank you for your kind comment. We additionally performed the plaque reduction neutralization test (PRNT) against SFTSV with positive sample by IFA to measure neutralizing antibody. Neutralizing antibody titer against SFTSV was 1:80. We added the method and result about PRNT against SFTSV in line 109-119 and 141-142.

Line 320-321: Please clarify the additional details of positive human serum against SFTSV and normal human serum, where did the sera come from? How are those sera identified?

→ Thank you for your nice comment. Serum from a patient with SFTS who was diagnosed by SFTSV PCR was used for positive control. For negative control, serum from a healthy donor without any history of infection with SFTSV was used. We revised the manuscript for clarification according to your comment (line 349-350).

Table 2: The flaviviruses (TBEV, WNV, DENV, and JEV) were tested positive at the sampling places Gangneung and Jeju, Is there cross identification between them?

→ So far, there were no domestic case reports of West Nile virus infection or dengue fever in South Korea. Whereas, Japanese encephalitis virus, a mosquito-borne flavivirus, is endemic in South Korea.

In this study, our primary aim was to investigate seroprevalence of TBEV and SFTSV in forest/field workers. However, we also analyzed anti-JEV IgG because of endemicity of JEV in South Korea and its possible cross-reactivity to TBEV and other flaviviruses. In addition, we analyzed anti-WNV IgG and anti-DENV IgG.

Peoples who have anti-DENV IgG or anti-WNV IgG were also positive for anti-JEV IgG in our study. Thus, it is possible that cross-reactivities of anti-JEV antibodies to DENV or WNV contribute to the seropositivity of anti-DENV IgG and anti-WNV IgG. However, it is not definite because we did not perform neutralization assays and PCR for DENV, WNV and JEV in this study. We added this point in the discussion section (line 193-202, 206-208).

Table 2: The JEV was detected positive in all the sampling places, have those people ever been bitten by ticks? Are there higher positive rates of JEV carried by ticks in those areas?

→ Japanese encephalitis virus, a mosquito-borne flavivirus, is endemic in South Korea. Japanese encephalitis vaccination was adopted as the national immunization program in South Korea since 1985. In a study on seroprevalence of neutralizing antibodies against JEV among the general population in South Korea, the seropositive rate was 97%-99% for the age of 20-29 years, 80%-89% in 30-49 years, and 75%-80% in 50-69 years.

In this study, our primary aim was to investigate seroprevalence of TBEV and SFTSV in forest/field workers. However, we also analyzed anti-JEV IgG because of endemicity of JEV in South Korea and its possible cross-reactivity to TBEV and other flaviviruses. We clarified this point in line 193-202.

Reviewer #3: Major

1. Authors describe “only 19 (3.3%) reported experience of tick bite” in line 113 and Seroprevalence of anti-TBEV IgG was 0.9% (5/583, 95% confidence interval [CI], 0.3-2.1) by ELISA. Neutralization assay for TBEV showed positive rate of 0.3% (2/583, 95% CI, 0.1-1.4) between line 116 and line 118.

Are 19 (3.3%), experience of tick bite, have seropositive of anti-TBEV IgG or positive of neutralization assay for TBEV?

The main transmission route of TBEV is tick bite. Therefore, could you describe about this in manuscript?

→ Thank you for your time and helpful suggestions about our manuscript. We revised our manuscript as your commented. Point-by-point responses to your review are described below. We really appreciate your positive consideration about our manuscript.

Anti-TBEV IgG or neutralizing antibodies against TBEV were not detected among 19 subjects who experienced recognized tick bite. However, there is a possibility that tick bite was not recognized. We added this point as a limitation of study (line 208-209).

2. Between line 124 and 124, authors describe below

“Among 583 subjects, positive rates for anti-DENV IgG and anti-WNV IgG were 0.5% (95% CI, 0.1-1.6). Peoples who have anti-DENV IgG or anti-WNV IgG also positive for anti- JEV IgG”

Antibodies of WNV have cross reaction with antibodies of JEV and gold standard of diagnosis of WNN is RT-PCR.

Is positive rate for anti-WNV IgG real positive for WNV?

→ It is possible that cross-reactivities of anti-JEV antibodies to DENV or WNV contribute to the seropositivity of anti-DENV IgG and anti-WNV IgG. However, it is not definite because we did not perform neutralization assays. In addition, PCR was not conducted in this study because the study population was not in acute phase of suspected infection and this study aimed to investigate the seroepidemiologic status of forest/field workers. We added this point as a limitation of study. In addition, we revised the discussion section as your commented (line 193-202, 206-208).

3. In line 127, authors describe “Only 1 forest worker in Yeongju was seropositive for SFTSV by IFA (Fig 2)” and the title (1:2,048) is relative high.

Could you do (real-time) RT-PCR about this specimen of this people?

Because atypical signs and symptoms and asymptomatic infections have also been identified and reported.

→ Thank you for your nice comment. We did not perform RT-PCR for SFTSV and we added this point as a limitation of study in line 206-208. However, we additionally conducted neutralization assay for SFTSV with the positive sample by IFA, and neutralizing antibody titer against SFTSV was 1:80. We added the method and result about PRNT against SFTSV in line 109-119 and 141-142.

Minor

In line 33, Could you write full name of ELISA?

→ We wrote full name of ELISA in line 32-33.

In line 34, Could you write abbreviation of indirect immunofluorescence assay?

→ We added abbreviation of indirect immunofluorescence assay in line 34.
