# Supplementary material for: Seroepidemiologic survey of emerging vector-borne infections in South Korean forest/field workers
Source: PLoS Negl Trop Dis. 2021 Aug 18;15(8):e0009687. doi: 10.1371/journal.pntd.0009687 (PMC8405005; doi:10.1371/journal.pntd.0009687)
Supplement: Attachment — Submitted filename: Responses to reviewers_PLOS Neglected Tropical Dis.docx [file pntd.0009687.s002.docx]

**Responses to reviewers**

**Reviewer #1**

Although this data could not show direct the evidence of TBEV, I agree this study could attribute to development of vaccine policy. I had several questions about result, but other reviewer already has been requested. So, I have resolved by author's answers.

→ Thank you for your time and efforts for the review of our manuscript. We really appreciate your positive consideration about our manuscript.

**Reviewer #3**

→ We really appreciate your positive consideration about our manuscript.

**Reviewer #4**

- While information on the testing details for TBEV and SFTSV are provided, there is no mention of the other diseases listed in lines 68-70 in the Introduction. It is good to know that these other viruses were also tested, so I believe there should be a brief description about their tests in the Methods (please see my comments also below under Results and Conclusions). Also, the authors may wish to reconsider the title (i.e. “emerging vector-borne infections”), since the authors focus their paper on TBEV and SFTSV…

→ Thank you for your time and helpful suggestions about our manuscript. We revised our manuscript based on your comments. Point-by-point responses to your review are described below. We really appreciate your positive consideration about our manuscript.

Human IgG against WNV, DENV, and JEV were measured using the Anti-West Nile Virus ELISA (EI 2662-9601G, Euroimmune), Anti-Dengue Virus ELISA (EI 266b-9601G, Euroimmune), and Anti-JEV ELISA (EI 2663-9601G, Euroimmune), respectively, according to the manufacturer’s protocol. We already described these assays in the Methods (Line 83-86).

As your comment, we focused our research on TBEV and SFTSV. Both are considered as important emerging vector-borne viruses regarding forest and field. Thus, we would like to keep current title of the manuscript. Your understanding about our situation will be appreciated.

- As stated for the Introduction and Methods sections, a brief statement about the negative results for the other viruses tested from the specimens of all 583 persons would be informative. Table 2 is presented, but it does not seem to be referenced in the main text; for Table 2, what the numbers presented represent should be clearly indicated (i.e. “n (%)”; this should be listed at the top, not under TBEV). Some of the details regarding these tests become clear only in the Discussion section (lines 198-202, 212-213)...

→ Seropositive rates for DENV, WNV, and JEV were described in the Result section (Line 139-142). Among 583 subjects, positive rates for anti-DENV IgG and anti-WNV IgG were 0.5% (95% CI, 0.1-1.6). Overall positive rate of anti-JEV IgG tested using ELISA was 15.8% (95% CI, 13.0-19.1).

As your comment, the format of the Table 2 has been reorganized. What the numbers mean was clearly indicated, adding the ‘Number of seropositive subjects (%)’.

- Table 1: Having this type of standard table 1 to show the demographic features of the study population is useful, but the presentation could be improved to help guide the reader. For instance, the years when these data were collected would be useful (2017-2018). In addition, the title heading (below “N=583”) could have “n (%)”, and an asterisk could be used to show where the data are “median (range)” (e.g., age, occupation duration, field worker duration). Also, for the Occupation category, it states that range is in “yr” but “1 month-50” appears, while the other says simply “1-60”, so this should also be standardized in presentation. And, form the text (lines 127-8), it appears that the “-“ were “0” (e.g., Vaccination history for yellow fever, past history of vector-borne diseases), so if that were the case, an explicit “0 (0)” is probably more appropriate. Also, for these data on vaccination histories and past history of diseases, if they were based on self-report, that should be explicitly indicated (as with the tick-bite experience).

→ Thank you for your kind comments. We revised the Table 1 as your comments. The years when these data were collected were added. Asterisk was used to show the data presented with median (range). We corrected 1 month to 0.08 year for standardization in presentation and revised “-” to “0 (0)” for clarification.

Data on vaccination history and past history of diseases were collected based on the self-report. We verified this point in Methods section (Line 78-79).

- Table 1 and Results (or Discussion): albeit self-report, it would be useful to know what the results were for these 19 persons with a tick bite (for instance, were they more likely to have tested positive for SFTSV or TBEV?)—if there was anything notable about the 19, perhaps that could be briefly commented (conversely, if none of the seropositive SFTSV or TBEV individuals reported a tick bite, that would also be worth mentioning).

→ Thank you for your comment. Among 19 persons with tick-bite experience, three (15.8%) were positive for anti-JEV IgG and all were negative for anti-TBEV IgG, anti-DENV IgG, anti-WNV IgG, and anti-SFTSV IgG. We added this point in Line 145-147.

- Line 169: as commented in the Introduction, can the authors be sure that the possible infection in China was asymptomatic (he may have had symptoms/signs but was not tested and diagnosed at the time)?

→ Thank you for your helpful comment. It is hard to confirm that the possible infection was asymptomatic or not. Therefore, we removed ‘asymptomatic’ to clarify.

- Line 177: as with the abstract, I would state as the number of “reported” cases since these are surveillance data.

→ We revised ‘the number of cases’ to ‘the number of reported cases’ as your recommendation (Line 27, 45, 181).

- Lines 222-224: while this is my personal view, I believe that the statement, “it is necessary to raise the awareness of physicians about TBEV infection and to establish a surveillance system in South Korea” is quite strong given what the study found and what the authors comment in their limitations. Perhaps additional special studies are needed before concluding that it is necessary to establish a surveillance system for TBEV (given the work necessary for setting up a public health surveillance system, including legal procedures, etc.)

→ Thank you. As your recommendation, we revised the sentence as follow: This study shows that it is necessary to raise the awareness of physicians about TBEV infection and to make efforts to survey and diagnose vector-borne diseases in South Korea (Line 40, 52, 227).

- The English was competent, but there were some minor issues (phrasing, verb tense, etc.) so a review of the English by a native scientific writer would make the paper more readable.

Some examples are listed below:

- Line 57: “Although existed long time ago”

→ We corrected the phrase into “Although existed long time” (Line 58).

- Line 107: “After washed”

→ We corrected the phrase into “After washing” (Line 109).

- Line 138-139: “Peoples who have anti-DENV IgG or anti-WNV IgG also positive for anti- JEV IgG.”

→ We corrected the phrase into “Peoples who have anti-DENV IgG or anti-WNV IgG were also positive for anti- JEV IgG” (Line 140).

- Line 190: “seropositive rate has decreased”

→ We corrected the phrase into “seropositive rate decreased” (Line 194).

- Line 203: “one serum which is positive by”

→ We corrected the phrase into “one of two positive sera by neutralization assay” (Line 207).

- Line 354: "foreast"

→ We corrected the word into “forest” (Line 358).

- The original article, "Seroepidemiologic survey of emerging vector-borne infections in South Korean forest/field workers" (PNTD-D-21-00010R1), is a straightforward report regarding a serological investigation of tick-borne encephalitis virus (TBEV) and severe fever with thrombocytopenia syndrome virus (SFTSV) infections among forest and field workers in South Korea. The sample was large with nearly six hundred persons, and the seroprevalence for TBEV was 2/583 (0.3%) and 1/583 (0.2%) for SFTSV. The authors recommend raising awareness of physicians about TBEV infection and to establish a surveillance system for TBE in South Korea.

A few general comments not listed in the above sections:

Abstract, Line 27: “…the number of cases has increased…” should probably be stated as “…the number of reported cases has increased…”, since these are passive surveillance data that are reported by healthcare workers (which are dependent on patients seeking care, the clinician suspecting disease, testing for the disease, and ultimately reporting the disease). This applies to other areas where this phrasing appears (e.g., Author summary, Discussion).

→ Thank you for your nice comment. We revised ‘the number of cases’ to ‘the number of reported cases’ (Line 27, 45, 181).

- Introduction, Line 71: while serologic surveys can be useful for detecting asymptomatic infections, given that the authors tested with IgG, I would think that past infections that went undetected/unreported would also be captured and offer valuable information. Do the authors really mean to limit their statement to "asymptomatic infections"? This applies to other areas where the emphasis is made on asymptomatic infections.

→ Thank you for your helpful comment. It is hard to confirm that the possible infection was asymptomatic or not. Therefore, we removed ‘asymptomatic’ to clarify.
